# The acceptance and impact of Google Classroom integrating into a clinical pathology course for nursing students: A technology acceptance model approach

**DOI:** 10.1371/journal.pone.0247819

**Published:** 2021-03-05

**Authors:** Tzu-Hao Huang, Fen Liu, Li-Chen Chen, Ching-Ching Tsai

**Affiliations:** 1 Department of Nursing, Chang Gung University of Science and Technology, Taiwan, R.O.C; 2 Department of Nursing, Chang Gung Memorial Hospital, Linkou, Taiwan, R.O.C; 3 Department of Pediatrics, New Taipei City Municipal Tucheng Hospital, New Taipei, Taiwan, R.O.C; 4 Division of Allergy, Asthma and Rheumatology, Department of Pediatrics, Chang Gung Memorial Hospital, Linkou, Taiwan, R.O.C; 5 College of Medicine, Chang Gung University, Taoyuan, Taiwan, R.O.C; 6 Department of Cardiology, Chang Gung Memorial Hospital, Linkou, Taiwan, R.O.C; KTH Royal Institute of Technology, SWEDEN

## Abstract

**Background:**

Google Classroom (GC) is a free web-based instructional platform rarely used for nursing student education. The acceptance, intention to use, and learning outcomes of GC remain unclear in Taiwan. We sought to identify the technology acceptance level and factors affection the intention to use GC. We also explored how integrating GC into traditional teaching affects learning satisfaction and academic achievement among nursing students in Taiwan.

**Methods:**

In this randomized controlled study, based on a technology acceptance framework, 74 nursing students were randomly assigned in clusters to experimental (n = 39) and control (n = 35) groups during the spring semester of 2018. In Weeks 3–18, each member of the experimental group received one hour of traditional and GC teaching per week. The control group received two hours of traditional teaching per week. Both groups were asked to complete questionnaires to evaluate learning satisfaction and academic achievement during weeks 10 (mid-semester) and 18 (end-of-semester). The experimental group additionally completed technology acceptance questionnaire*s* in both situations.

**Findings:**

In the experimental group, the overall end*-*of-semester technology acceptance score was high (141.8 out of 155); their perceived ease of use, intention to use, and technology acceptance scores increased significantly compared to mid-semester (*p*<0.05). At the end-of-semester, perceived playfulness and perceived usefulness explained 63.5% of the variance in intention to use. Regardless of whether the assessment was administered mid-semester or at the end-of-semester, the experimental group had higher learning satisfaction and academic achievement scores than the control group. However, the degree of progress on learning satisfaction and academic achievement demonstrated no significant between-group differences.

**Conclusions:**

The experimental group demonstrated high acceptance of GC. Playfulness and usefulness positively influenced nursing students’ intention to use GC. Blended learning—in combination with GC and traditional methods—resulted in similar learning satisfaction and academic achievement when compared to traditional learning. More research is needed to explore the effectiveness of blended learning through the GC platform with different courses and ethnic groups.

## Introduction

Clinical pathology is a fundamental component of nursing education, and a basic medical science discipline on Taiwan’s nurse licensing exam for nurse practitioners. Students typically consider such courses difficult-to-understand and unappealing and often perform poorly on licensing exams [1]. Basic medical science courses are predominantly taught through traditional methods—including teacher-centered approach, knowledge-based, in-class, and face-to-face lecture formats—that limit attention, engagement, and application. This may impair a student’s ability to learn [1–3].

Rapid technological advancement has promoted the substitution of traditional learning with e-learning [4]. E-learning is an educational method that uses new information technology and internet communication to promote learning [5, 6]. The goal is to provide students with a student-centered, enjoyable, and interactive learning environment. E-learning conveys benefits from its rapid delivery, flexibly, and ability to adapt to varying formats. The transformation of knowledge-integration into clinical practice may improve the quality of education [5, 6]. However, e-learning requires additional research, the development of new materials by educators, increases in broadband accessibility, and user-friendly technology to increase students’ self-discipline and improve the interactions between students and teachers. Unfortunately, tracking students’ learning outcomes can be more difficult with e-learning than with traditional learning [5, 6].

E-learning has different models that have been used in practice, including as an accompaniment to traditional face-to-face learning, integration of face-to-face learning with online learning, and pure online learning [5]. Although many students have a positive attitude towards e-learning, some argue that e-learning should complement rather than replace traditional learning, especially since face-to-face learning settings provide students and teachers opportunities to interact physically and emotionally [7]. There are many factors that influence both e-learning and traditional learning. For example, something that supports blended learning, or uses blended teaching, becomes an alternative method for overcoming the drawbacks of traditional and e-learning [3, 8]. Blended learning combines e-learning and traditional methods. Blended learning methods are widely accepted in educational fields around the world, and allow teachers to transform from an instructor of knowledge to a facilitator. This provides students with more fascinating and effective instruction, catered to their own needs, which has diminished instructor costs, while increasing student performances and experiences [9]. This suggests that blended learning may be a suitable medium for a clinical-based pathology course [2, 10].

The teachers’ initial challenge involves learning to use an e-learning management system as a tool for blended learning. At our school, each teacher or student is given a personal G-mail account after enrolling and provided access to the Adobe e-campus, which has supported teaching and learning activities since 2009. The e-campus can be connected to the School Affairs Information System, which provides paperless communication between teachers and student services. Users can select historical or current courses from the main page. Each course’s main page contains class information, materials, scores, and tools. Teachers can publish news, upload lecture notes/files/homework, view and/or grade homework, and announce scores. Students can download course materials, upload or download homework, and ask the teacher questions after class. The system can send e-mails to notify course members of messages automatically or manually. Most of the courses in our school use e-campus as a teaching platform to provide students with one-stop service, easily share learning materials, and ask questions without time constraints [11]. However, many students rarely used e-campus. These students had an average of only 8.5 logins per semester. The stated disadvantages to e-campus were limited storage volume and functions, inability to link to videos or news URLs, use of other applications, and inability to interact with students in real-time. Considering its speed, stability, functionality, accessibility, technical user-friendly, and low-cost, Google Classroom (GC) may emerge as a suitable learning platform [12].

Google software can be used on desktop computers, laptops, tablets, and mobile phones [12]. GC has been available for use by educational institutions since August 2014 [13]. GC is free of spatio-temporal constraints, requires no maintenance after setup, and allows easy development of course content through access/integration with Google’s online services/features (e.g., Google Drive, YouTube, News, Google Docs) and other applications. Tests and assignments can be created in various formats, instantly organized, and graded. Online discussion boards allow students to easily interact with peers/teachers in a real-time environment [12, 14]. However, the problems students faced using GC are similar to those associated with other learning management systems such as internet speed and coverage, difficulty understanding platform operation, the need to spend more time practicing and studying, and under-utilization [12, 15]. To build, implement, and motivate students to use the new learning platform, we adopted the technology acceptance model (TAM) as the research framework to design educational materials and assess students’ acceptance and use of GC [16–18].

The TAM was developed by Davis [19] and based on reasoned action and planned behavior theories. It explores the predictors of a user’s potential acceptance/rejection of new technology and reflects their willingness to use the technology [19, 20]. The original TAM encompassed five components—perceived usefulness, perceived ease of use, attitude toward using, intention to use, and actual use [19, 20]. Past studies rarely included all five components as variables; perceived usefulness, perceived ease of use, and intention to use are the most commonly used components. The most important predictors of intention to use are perceived usefulness and perceived ease of use [21]. Perceived usefulness denotes whether individuals believe technology can improve learning performance; perceived ease of use refers to whether individuals believe technology can be used effortlessly; intention to use refers to the individual’s levels of intent to use technology [19, 20]. Over the last three decades, many scholars have modified TAM (e.g., TAM 2, TAM 3, and extensions of TAM) because perceived usefulness and perceived ease of use are influenced by many external variables; they may affect the intention to use technology together. The modified content can be divided into four main categories: external predictors (prior usage experience, self-efficacy, playfulness), factors from other theories (trust, user participation), contextual factors (sex, culture), and usage measures (usage perception, attitude toward technology) [22]. Among more than 100 external variables that affect perceived usefulness and perceived ease of use, the most discussed are interaction, self-efficacy, and playfulness [21]. The interaction involves individuals’ interactive/cooperative learning with their peers and teachers. Self-efficacy involves individuals’ perceptions of their ability to learn by using technology. Playfulness involves concentration, curiosity, and enjoyment associated with technology use [21, 23, 24].

There are no standard methods used in blended learning models [9]. Different types of blended learning affect student learning outcomes [25]. Teachers usually through affective (such as satisfaction, and self-efficacy), cognitive (such as knowledge and problem-solving abilities), and psychomotor (such as clinical skills) domains to evaluate the learning outcomes of students after implement blending teaching [26]. Blended learning inconsistently improves learning outcomes. Blended learning enhances students’ learning outcomes and overall satisfaction [27, 28]. A meta-analysis indicated that blended learning could improve nursing students’ satisfaction and knowledge more effectively than traditional learning [7]. Nevertheless, other studies showed that blended learning did not significantly improve nursing students’ satisfaction and/or knowledge compared to traditional learning [2, 29]. Further, meta-analyses have revealed that blended learning does not generate consistently positive student satisfaction and knowledge [30, 31]. The inconsistency of these findings may be the result of various learning methods, contents, situations [32], students’ motivation, expectations, self-directed learning readiness [5, 29], and discipline-specific differences [31]. Besides, curriculum design, teaching platform, teaching methods, and professional image all affect students’ satisfaction and learning performance [5, 8, 29].

Previous studies have applied GC to nursing programs [15, 33–36] whose students demonstrated a high level of satisfaction with GC. However, these studies employed exploratory [15, 33] or pre-experimental designs [34–36] for a small number of samples. There has been very little research on the use of GC with nursing students in Taiwan, and limited evidence exists to support the use of blended learning for the basic medical science course taken by nursing students [2, 27]. Blended learning can be challenging because it requires teachers prepare (and apply) a new teaching method, especially during the initial implementation period. We must identify which method—traditional or blended—is more effective for teaching undergraduate nursing clinical pathology courses.

The objectives of this study were: 1. to determine if nursing students would accept GC and what factors influenced students’ intention to use GC; and 2. to examine the effects of integrating GC with traditional teaching on learning satisfaction and academic achievement. An experimental group was used to examine the first objective and answer the two following research questions. 1. What is the acceptance level of GC? 2. What factors affected students’ intent to use GC at the end of the semester? We hypothesized that blended learning would produce non-inferior results when compared to traditional learning.

## Materials and methods

### Participants

This randomized controlled study was conducted after approval from the Institutional Review Board of Chang Gung Medical Foundation (IRB No. 201702002A3). Five-year junior college program, fourth-grade nursing students at a university of science and technology in Taiwan participated. Inclusion criteria were: 1. enrolment in the clinical pathology course; 2. signing of a consent form by each student or his or her legal representative after receiving a full explanation of the study. We excluded students who did not complete the course. In Week 1, the non-teaching researcher explained the research objectives and procedure to the two classes separately, stressing that they could withdraw from the study anytime without any justification. Students were told that non-participation or dropping out of the study would not affect their right to complete the course. Students younger than 20 years were required to obtain their legal representative’s signature on the consent form. Participation was voluntary and all participants provided written informed consent.

Referencing a previous study that compared GC and traditional teaching, and the effects of these methods on teaching efficiency and academic achievement in college students, the effect size (ES) was 0.14–0.93 with an average of 0.67 [37]. Accordingly, a sample size of 72 subjects (36 per group) was calculated using G power 3.1 software (for a two-tailed independent samples t-test, a power of 0.8 and an α of 0.05). In the spring semester of 2018, 90 students enrolled in the course and were divided into two classes by the Academic Affairs Office. Before the start of the semester, they were clustered and randomly assigned to either the experimental or control group by a non-teaching researcher. Sixteen students/legal representatives did not complete the consent form. In total, 74 participants were enrolled (experimental group = 39; control group = 35), for a participation rate of 82.2%. All participants completed the course (S1 Fig).

### Teaching interventions

The clinical pathology course was a two-credit (2 hours/week; 18 weeks) specialized elective course, which including 12 units of clinical microbiology; clinical immunology; routine bloodwork; cardiovascular, hematology, respiratory, digestive, metabolic, and urinary system disease; liver and renal function tests; and tumor marker inspection. The course units and their corresponding materials were the same for both groups. Two weeks before the course began the teacher uploaded the syllabus and schedule on the e-campus. Before class every week, the teacher uploaded lecture notes and reference materials to the e-campus, where all students were able to download the materials. The same experienced teacher, with over 20 years of teaching experience, instructed both groups of students. This was done so as to mitigate any potential teacher-related effects.

During traditional teaching, the teacher used PowerPoint to deliver lecture note content in a face-to-face manner. At times, the teacher would play various YouTube news and related videos in the classroom to support the students’ coursework. Students were also encouraged to study the reference materials uploaded by the teacher (such as national examination papers) after class. Over the course of the semester, students were able to ask the teacher questions in class or through e-campus after class. The control group received two hours/week of traditional teaching. The experimental group received one hour/week of traditional teaching and one hour/week of GC-integrated activities.

We used the TAM model to design and implement teaching within GC. During the setup phase we developed the GC platform content for 12 units of a clinical pathology course. Each unit included separate “zones” for Videos, News, Games, Quizzes, and Arena. The Video zone linked to teacher-made or YouTube videos; the News zone linked to Google News or the news section of YouTube to integrate pathological knowledge with everyday life and make knowledge feel more useful. The Games zone delivered course content using single or multiplayer games with time-based leader boards, through the applications Quizlet, Quizizz, and Quizalize, which helped convert learning into a more playful process, while also reinforcing memory. The Quizzes zone provided access to past national examination papers by linking to Google Drive, and used Google Forms to conduct online tests and give instant feedback, thus achieving the learning objectives using brainstorming and critical thinking. The Arena zone allowed students to compete with each other to answer questions raised by the teacher, engage in discussions, or challenge one another. These activities were followed by the teacher’s commentary, thus strengthening teacher-student interactions [38].

During the implementation phase, we found that all students in the program had internet-enabled mobile devices. The students were allowed to bring their devices to school during the first week and used their own devices to access the GC platform at any time for repeat and independent learning and to ease the transition to the new platform. During week 2, the teacher told students how to download the GC application and invited them to log into the course. Each student had a personal G-mail account after enrolling. Therefore, there was no need to register an e-mail account for GC. The teacher introduced GC and discussed its usage and functions. During Weeks 3–18, the experimental group received one hour/week of GC-integrated activities, including 10- to 15-minute activities in each of five themed zones which were Videos, News, Games, Quizzes, and Arena. The teacher addressed problems or difficulties encountered by students as they arose and ensured they could use the GC platform in the classroom. We thought that if a student believed in his or her own ability to use the GC, he or she might demonstrate increased self-efficacy. Students could review or ask questions anytime and anywhere after class. For students who did not have unlimited internet access, free wireless internet was provided on campus or in the dorms.

### Measures of variables

We used an original basic information form to collect participants’ demographic data. This questionnaire collected information on sex, age, weekly frequency of internet use, daily hours of internet use, and household devices compatible with e-learning (e.g., desktop computers, tablets, laptops, and smartphones).

The questionnaire on technology acceptance (TA) of GC devised by Wu [24] was used to measure the experimental group’s acceptance of GC. We used the TAM-based questionnaire as a development framework to explore the relationship between external variables (including interaction quality, computer self-efficacy, and perceived playfulness), perceived usefulness, perceived ease of use, and intention to use GC. The questionnaire consists of 31 items across six dimensions: interaction quality (six items), computer self-efficacy (five items), perceived playfulness (five items), perceived usefulness (five items), perceived ease of use (five items), and intention to use (five items). Each item was scored on a five-point Likert scale, where 1 = strongly disagree, 2 = disagree, 3 = general, 4 = agree, 5 = strongly agree. Higher scores implied a more positive attitude toward the relevant dimension. The original questionnaire generated Cronbach’s α values that ranged between 0.89–0.97. The expert and discriminant validity indicated satisfactory discriminating power and homogeneity of the items [24]. We derived a total score of 31–155 from the sum of scores across the six subscales [39]. Higher total scores represented higher overall TA levels of GC. Cronbach’s α in the current study was 0.80–0.95. Additionally, we asked respondents to answer the question “How do you feel about using GC?” at the end-of-semester in order to assess the experimental group’s perceptions related to GC.

The learning satisfaction questionnaire developed by Cheng and Cheng [40] was used to measure students’ learning satisfaction. The original questionnaire was used to assess college students’ satisfaction with the core curriculum. With the consent of the questionnaire’s author, we added the name of the clinical pathology course to the otherwise-unaltered questionnaire. The full questionnaire consists of 21 items across three subscales: procedure and interaction (n = 7), encouragement and benefit (n = 8), and concern and openness (n = 6). The items are scored on a five-point Likert scale ranging from: strongly dissatisfied (1); dissatisfied (2); neutral (3); satisfied (4); to strongly satisfied (5). The total score was calculated for each participant by summing scores across the three subscales (range: 21–105). Higher total scores indicated higher overall learning satisfaction. The original questionnaire generated a Cronbach’s α value of 0.92–0.98 and a two-week test-retest correlation coefficient of 0.72–0.83; exploratory factor analysis showed that the total variance explained by the three subscales was 73%. These values indicated satisfactory reliability and validity [40]. Cronbach’s α was 0.87–0.95 in the current study.

A test, self-developed by the teacher for this study and comprised of single-answer questions, was used to evaluate students’ academic achievement while acquiring subject-specific knowledge. Two 40-item tests were conducted (during mid-semester and end-of-semester periods). Each item received 2.5 points, with a total score ranging from 0–100. Higher scores denoted higher levels of academic achievement. The mid- and end-of-semester tests were based on the learning objectives of each unit taught during the study period. The test questions were checked for clarity by a senior nursing teacher (non-teaching researcher).

### Data collection

All data were collected and analyzed by a non-teaching researcher, and the teaching researcher did not alter, in any way, the data collection or analysis. During Weeks 1–2, both groups received traditional teaching; as university rules state, students are allowed to join and/or withdraw from courses within the first two weeks. During Weeks 3–18, the experimental group received traditional and GC teaching, whereas the control group only received traditional teaching. Both groups were asked to complete the learning satisfaction questionnaire and the self-developed questionnaire during week 10 (mid-semester) and week 18 (end-of-semester). The experimental group was required to complete an additional TA questionnaire at both timepoints. Given the control group’s right to learn, after completing the end-of-semester learning satisfaction questionnaire and self-developed questionnaire, the teacher invited the control group to the GC course platform and informed them of its features and use. The teacher also continued providing support to students and guidance regarding the platform after course completion.

### Data analysis

SPSS 23.0 (IBM Corporation, U.S.) statistical software was used for data analysis. Descriptive statistics included the number of people and percentage for internet and device-use related characteristics, means, and standard deviations (SDs) for TA scores, learning satisfaction, and academic achievement. Q-Q and scatter plots were used to examine the distributions of continuous variables. All data demonstrated a normal distribution and therefore met the assumption of normality necessary for parametric tests. Inter-group differences in basic characteristics were measured using the Chi-square test (or Fisher’s Exact test, if the expected value was <5) and an independent samples t-test.

TA was analyzed conducted in the experimental group only. The paired-samples t-test to compare the mid-semester and end-of-semester TA scores to understand acceptance of GC over time. Univariate analyses were performed, using simple linear regression to measure the effects of basic characteristics on the intention to use scores. Pearson’s correlation was used to examine the relationships among the TA subscales. With the end-of-semester intention to use score as the dependent variable, independent variables with a significance of *p*<0.05 during the univariate analysis were entered into a forward stepwise linear regression analysis. The purpose of this test was to identify the factors that influenced nursing students’ intention to use GC at the end-of-semester.

To investigate the intervention’s effect, an interaction term of a generalized estimating equation (GEE) was used to identify any significant inter-group differences between the mid-semester and the end-of-semester scores. An intra-group comparison between mid-semester and end-of-semester scores was carried out using paired-samples t-tests, followed by an inter-group comparison of mid-semester and end-of-semester scores via independent samples t-tests. We calculated Hedges’ g, a measure of ES, for each independent and paired samples t-test. A small effect was less than 0.5, a medium effect ranged from 0.5 to 0.8, and a large effect was greater than 0.8 [41]. Two-tailed tests were adopted, with statistical significance defined as *p*<0.05.

## Results

### Basic characteristics in participants

Of the 74 participants whose data were analyzed, the average age was 19.1 years, 93.2% were female, the average frequency of internet use was >6 days/week, and 74.3% used the internet for >3 hours/day. There were no significant inter-group differences in basic characteristics (*p*>0.05; Table 1).

**Table 1:** Inter-group comparison of basic characteristics.

	Total (n = 74)	Experimental (n = 39)	Control (n = 35)	
Item	n (%)	n (%)	n (%)	*p*
Sex^a^				1.00
Female	69 (93.2)	36 (92.3)	33 (94.3)	
Male	5 (6.8)	3 (7.7)	2 (5.7)	
Household device^a^				1.00
No	9 (12.2)	5 (12.8)	4 (11.4)	
Yes	65 (87.8)	34 (87.2)	31 (88.6)	
Dorm device^b^				0.07
No	30 (40.5)	12 (30.8)	18 (51.4)	
Yes	44 (59.5)	27 (69.2)	17 (48.6)	
Mobile device^a^				1.00
No	1 (1.4)	1 (2.6)	0	
Yes	73 (98.6)	38 (97.4)	35 (100)	
Web-enabled cell phone^a^				1.00
No	3 (4.1)	2 (5.1)	1 (2.9)	
Yes	71 (95.9)	37 (94.9)	34 (97.1)	
Frequency of internet use (weekly) ^a^				0.47
3–5 days	1 (1.4)	0	1 (2.9)	
>6 days	73 (98.6)	39 (100)	34 (97.1)	
Hours of internet use (daily) ^a^				0.89
1–2 hours	1 (1.4)	1 (2.6)	0	
2–3 hours	18 (24.3)	10 (25.6)	8 (22.9)	
>3 hours	55 (74.3)	28 (71.8)	27 (77.1)	
	Mean ± SD	Mean ± SD	Mean ± SD	
Age^c^	19.1 ± 0.4	19.1 ± 0.3	19.2 ± 0.4	0.07

^a^ denotes Fisher’s exact test

^b^ denotes Chi-square test

^c^ denotes independent samples t-test

### Technology acceptance of Google Classroom

After integrating GC, the mid-semester and end-of-semester overall TA scores of the experimental group were 136.7 ± 13.3 and 141.8 ± 12.1 (out of 155), respectively. The average end-of-semester scores for perceived ease of use, intention to use, and overall TA were significantly higher than the mid-semester scores. The ES was 0.11–0.40 (Table 2; *p<*0.05).

**Table 2:** Comparison of technology acceptance within the experimental group between mid-semester and end-of-semester (n = 39).

Item	Mid-semester	End-of-semester		
	Mean ± SD	Mean ± SD	*p*	Effect size
Interaction quality	26.4 ± 2.7	27.3 ± 2.4	0.08	0.35
Computer self-efficacy	22.3 ± 2.6	23.2 ± 2.3	0.06	0.36
Perceived playfulness	21.3 ± 3.2	22.1 ± 2.9	0.06	0.26
Perceived usefulness	22.7 ± 3.0	23.0 ± 2.3	0.44	0.11
Perceived ease of use	22.6 ± 2.5	23.6 ± 2.4	0.04*	0.40
Intention to use	21.4 ± 3.0	22.5 ± 2.7	0.04*	0.38
Overall technology acceptance	136.7 ± 13.3	141.8 ± 12.1	0.02*	0.39

Note: Paired-samples t-test.

*p<0.05.

On average, females scored significantly higher than males for intention to use (S1 Table). All six scales of TA were moderately-to-strongly positively correlated (*r* = 0.34–0.76, *p*<0.05), with a strong positive correlation between perceived playfulness and intention to use (*r* = 0.76, *p*<0.01; S2 Table). For stepwise linear regression analysis, the independent variables were sex, interaction quality, computer self-efficacy, perceived playfulness, perceived usefulness, and perceived ease of use, and each variable’s variance inflation factor was <2.5. This indicated that the responses satisfied the no-collinearity assumption. Stepwise linear regression indicated that perceived playfulness (*β =* 0.51, *p<*0.001) and perceived usefulness (*β =* 0.38, *p<*0.01) explained 63.5% of the variance in intention to use GC at end-of-semester (Table 3).

**Table 3:** Multiple stepwise regression analysis of intention to use for the experimental group at the end-of-semester (n = 39).

	Model 1					Model 2				
	B	*β*	SE	95% CI for B	t	B	*β*	SE	95% CI for B	t
Constant	6.79		2.24	2.25–11.32	3.03**	1.71		2.71	-3.77–7.20	0.63
Perceived playfulness	0.71	0.76	0.10	0.51–0.91	7.08***	0.47	0.51	0.12	0.22–0.72	3.83***
Perceived usefulness						0.45	0.38	0.16	0.13–0.77	2.87**
F	50.06***					34.04***				
R^2^	0.575					0.654				
Adjusted R^2^	0.564					0.635				
R^2^ Change	0.575					0.079				

Note: Independent variables were sex, interaction quality, computer self-efficacy, perceived playfulness, perceived usefulness, and perceived ease of use (end-of-semester).

***p<0.001

**p<0.01.

### Effects on learning satisfaction and academic achievement

Table 4 illustrates the learning satisfaction and academic achievement scores of both groups. The average scores of the experimental group were higher than those of the control group for all subscales, but only significantly higher for the mid-semester learning satisfaction subscale of procedure and interactions (33.8 ± 1.9 vs. 32.6 ± 2.7, ES = 0.51, *p* = 0.03); GEE revealed no significant inter-group differences between the mid-semester and end-of-semester changes in learning satisfaction and academic achievement scores. Univariate analysis indicated that the experimental group did not differ significantly between mid-semester and end-of-semester in overall learning satisfaction and its subscales. The ES range was 0.18–0.41. End-of-semester scores of the control group for encouragement and benefit subscale, and overall learning satisfaction were significantly higher than their mid-semester scores. The ES range was 0.36–0.40. The average end-of-semester academic achievement score of both groups improved significantly compared to their mid-semester academic achievement score (*p*<0.01).

**Table 4:** Inter-group comparison of learning satisfaction and academic achievement.

	Experimental	Control			Time * Group		
Item	(n = 39)	(n = 35)	*p*^*b*^	ES	B	SE	*p*^*c*^
Procedure and interaction							
Mid-semester	33.8 ± 1.9	32.6 ± 2.7	0.03*	0.51	-0.99	0.61	0.11
End-of-semester	33.4 ± 2.5	33.2 ± 2.3	0.71	0.08			
*p*^*a*^	0.41	0.16					
ES	0.18	0.23					
Encouragement and benefit							
Mid-semester	36.2 ± 3.6	35.1 ± 4.5	0.26	0.27	-0.54	1.00	0.59
End-of-semester	37.3 ± 3.5	36.8 ± 3.8	0.53	0.14			
*p*^*a*^	0.10	0.03*					
ES	0.30	0.40					
Concern and openness							
Mid-semester	27.7 ± 2.6	26.8 ± 3.1	0.16	0.31	-0.02	0.67	0.98
End-of-semester	28.7 ± 2.1	27.7 ± 2.3	0.07	0.45			
*p*^*a*^	0.08	0.05					
ES	0.41	0.32					
Overall learning satisfaction							
Mid-semester	97.7 ± 7.3	94.5 ± 9.5	0.11	0.38	-1.54	2.01	0.45
End-of-semester	99.4 ± 7.4	97.7 ± 8.0	0.35	0.22			
*p*^*a*^	0.26	0.03*					
ES	0.23	0.36					
Academic achievement							
Mid-semester	79.7 ± 14.1	77.6 ± 10.1	0.46	0.17	-0.09	1.97	0.97
End-of-semester	87.8 ± 8.4	85.8 ± 10.5	0.37	0.21			
*p*^*a*^	<0.01**	<0.01**					
ES	0.68	0.78					

^a^ denotes paired-samples t-test

^b^ denotes independent samples t-test

^c^ denotes generalized estimating equations analysis—time (end-of-semester vs. mid-semester) × group (experimental vs. control).

Abbreviations: ES = effect size.

*p<0.05

**p<0.01

## Discussion

Our results support the application of GC for clinical pathology course among nursing undergraduate students in Taiwan. The experimental group demonstrated high acceptance of GC, and students’ perception of playfulness and usefulness positively influencing the intention to use GC. GC merged with traditional teaching was feasible and non-inferior to traditional teaching.

### Technology acceptance of Google Classroom

Our first research objective was to determine if nursing students would accept GC and what factors influenced students’ intention to use GC. The total score, average mid-semester score, and average end-of-semester score for TA were 155, 136.7, and 141.8, respectively. The results indicated that GC was highly accepted by experimental group students, consistent with the previous studies [15, 42, 43].

Most students felt the GC usable, flexible, accessible, fun, time-saving, enhance their understanding, and hoped extended to other courses [15, 42, 43]. In this study, most students also have a positive perception about using GC, representations that “*I think GC is a very good application*. *The platform provides tests so that we can review after class and use it to check whether we understand after reading*.” (24#); “*I can easily use a mobile phone to practice the questions in the spare time and open the software when I am bored*.” (#4); “*I think it is very convenient to use this program in class; I can practice the exercises at any time and communicate with the teacher in a timely manner*.” (#18); “*Using the GC allows us to learn by playing games*, *that makes class more interesting*!” (#26); “*GC is very useful to improve grades and hope to promote it to other courses*.” (#16 and #24); “*GC provides a lot of useful information and links*, *which are highly practical*.” (#2). However, a small number of students have a negative view, states that “*I hope to use GC in the real classroom only*, *and don’t like to spend more time after class*.” (#5); “*At first I feel it interesting*, *but later I find it troublesome*.” (#11). In addition, some students had to familiarize themselves with the platforms [42]. A student said that “*I think GC is a bit complicated*.” (13#). The results of this study showed the perceived ease of use and intention to use scores at end-of-semester were significantly higher than at mid-semester. With increased use, students became more familiar with GC, found it easier to use, and became significantly more intent on using it in the future. This suggests that students need sufficient time to learn how to operate the software and become familiar with it. However, the exact definition of “sufficient time” requires future research.

Sex had a moderating impact on the relationship between perceived ease of use, perceived usefulness, self-efficacy, and intended use of the e-learning system [44]. Specifically, males exhibited lower intention to use scores than females after using GC. However, since our study had a relatively small sample of male participants, further investigations are warranted. Two meta-analyses revealed that the causal relationships between TAM variables were medium- to large-magnitude. The strength of these relationships was influenced by user type (students or teachers), questionnaire type (printed or online), freedom of technology adoption (voluntary or involuntary), and type of e-learning technology [18, 45]. Our results showed that interaction quality, computer self-efficacy, perceived playfulness, perceived usefulness, and perceived ease of use were significantly positively correlated with students’ intention to use GC, similar to previous studies [24, 46, 47]. Kumar and Bervell [48] used a modified version of the Unified Theory of Acceptance and Use of Technology 2 (UTAUT2) to establish models of students’ acceptance of GC; the results explained 63% of usage intentions, similar to the results of the current study. We identified perceived playfulness and usefulness as important predictors of intention to use. Specifically, perceived playfulness explained 56.4% of the variance of intention to use. This was possible because the GC platform was linked with the gamified educational software of Quizlet, Quizizz, and Quizalize, and included course-related competitive, time-limited, and interactive games. These components likely stimulated a high level of interest and perceived playfulness among the students, which may ultimately have enhanced their intention to use.

### Effect on learning satisfaction and academic achievement

Our second research objective was to examine the effects of integrating GC with traditional teaching on learning satisfaction and academic achievement. The clinical pathology course in this study did not assess psychomotor outcomes (such as performing specific clinical or operational tasks) through either its teaching content or objectives. Therefore, the outcomes we assessed were affective (such as satisfaction with the learning process) and cognitive (such as academic achievement) in nature. Our results supported our stated research hypothesis by indicating that blended learning produced non-inferior results to traditional learning. This finding concurs with two prior meta-analyses [30, 31] and independent studies of nursing students [2, 29].

The outcomes of blended learning are closely related to the teaching methods and environment, content and materials design, disciplines, teachers’ professional image, and students’ ability to self-direct and motivation [5, 8, 21, 29, 32]. Although the experimental group had higher average learning satisfaction and academic achievement scores than the control group, the inter-group differences were not significantly different, possibly because of many causes. At first, both groups received some traditional teaching, and the control group was also exposed to technological learning, such as the use of YouTube news/related videos in class. After then, the teacher had over 20 years of traditional teaching experience, was familiar with the course content. His traditional classroom also performed well in the previous course evaluations (scoring 4.3–4.8 out of 5) and has been highly favored by students. Finally, we think a ceiling effect inhibited the experimental group’s progress toward the end of the semester [49]. The experimental group’s average overall learning satisfaction score was 97.7 (out of 105) at midterm, and 99.4 by the end of the semester, even though more pronounced improvement was expected. In contrast, the control group scored 97.7 in overall learning satisfaction at the end of the semester, similar to the experimental group’s midterm score. Since the learning satisfaction levels progressed more slowly for the control group, they had greater room for progress after the midterm. In addition, a meta-analysis showed that blended learning affected academic achievement with an average ES of 0.59 (95% CI = 0.43–0.71) [50]. Our results showed that the experimental group’s academic achievement scores were higher than the control group; however, this difference did not rise to the level of statistical significance (ES = 0.37–0.46) and was slightly lower than the previous meta-analysis result. This may be related to the different disciplines, course content, tests types, and comparative teaching modes used in the two investigations.

### Limitations and suggestions

To our best knowledge, this was the first randomized controlled study to incorporate GC into nursing education. However, some limitations warrant consideration. This study had a small sample size from which to draw conclusions and future, well-powered studies are warranted. We were unable to validate the actual conditions of GC usage—nor other potential external variables such as personal learning style or attitude toward technology. All participants attended the same school and were enrolled in the same department. The students had many opportunities to communicate by other means, which could have contaminated our results. This study was conducted based on an elective course. Students attention to, or interest in, this course may differ from other courses and affect the learning outcomes of nursing students. Local measurement tools with limited reliability and validity may cause measurement bias. Finally, the study was conducted in a single university of science and technology in Taiwan and measured learning outcomes solely based on learning satisfaction and academic achievement. Thus, the results probably cannot be extrapolated to other universities and outcome indicators, and especially to different languages and cultures. Future studies should broaden the scope to different types of courses, institutions, and more diverse learning outcome indicators, such as learning motivation and attitude.

In the era of rapid technological advancement, many new e-learning management systems—including GC—add additional flexibility to traditional teaching methods. Teachers are often asked to learn to use new technology and will often prepare teaching materials from various digital sources, especially during the initial period [3]. The new blended teaching strategy using the GC platform is challenging for both teachers and students. Students’ views on the use of GC in this study will provide a useful reference for educators. The key to the successful application of GC lies in teachers’ ability to design and construct a teaching environment which maximizes the playfulness and usefulness of the platform to fully leverage their role as teacher and guide. Students were accepting of GC and reported feeling the platform had good functionality, accessibility, technicality, and mobility. Blended learning also reduces the time teachers spend delivering oral lectures. Students felt the combination of live learning and GC was enjoyable, thereby reducing the possibility of platform under-utilization. However, attention should be paid to concerns surrounding privacy, data protection, and users’ intellectual rights, especially on free platforms [12]. Blended and traditional teaching have similar learning outcomes. This implies that students can learn via new and different methods. Although we were fortunate to have access to good internet and mobile device support, barriers such as poor motivation and inability to operate the learning platform remain [5]. Educators should develop and optimize a "student-centered" teaching method which provides additional time to explore and learn to operate the new education platform.

## Conclusion

Most students were accepting of GC. Enhancing the playfulness and usefulness of GC will increase students’ intention to use. Blended learning—GC merged with traditional learning—was feasible and produced similar effects on learning satisfaction and academic achievement compared to the traditional learning model of clinical pathology for undergraduate nursing students. The free GC learning management platform can be integrated into traditional teaching to provide teachers and students with alternative learning options.

## Supporting information

S1 Fig Participation flowchart. (DOCX)

S1 Table Simple regression analysis of the experimental group’s intention to use Google Classroom at the end-of-semester. (DOCX)

S2 Table Pearson correlations between subscales of technological acceptance for the experimental group at the end-of-semester. (DOCX)

S1 File Data set. (XLSX)
