# Supplementary material for: The acceptance and impact of Google Classroom integrating into a clinical pathology course for nursing students: A technology acceptance model approach
Source: PLoS One. 2021 Mar 5;16(3):e0247819. doi: 10.1371/journal.pone.0247819 (PMC7935261; doi:10.1371/journal.pone.0247819)
Supplement: S1 Fig — (DOCX) [file pone.0247819.s001.docx]

S1 Fig. Participation flowchart.
