# Supplementary material for: The acceptance and impact of Google Classroom integrating into a clinical pathology course for nursing students: A technology acceptance model approach
Source: PLoS One. 2021 Mar 5;16(3):e0247819. doi: 10.1371/journal.pone.0247819 (PMC7935261; doi:10.1371/journal.pone.0247819)
Supplement: S1 Table — (DOCX) [file pone.0247819.s002.docx]

S1 Table. Simple regression analysis of the experimental group’s intention to use Google Classroom at the end-of-semester (n=39).

| Item | No. | mean ± SD | *p* |
| --- | --- | --- | --- |
| Sex |  |  | 0.04* |
| Female | 36 | 22.8 ± 2.7 |  |
| Male | 3 | 19.3 ± 1.2 |  |
| Household device |  |  | 0.19 |
| No | 5 | 24.0 ± 1.7 |  |
| Yes | 34 | 22.3 ± 2.8 |  |
| Dorm device |  |  | 0.44 |
| No | 12 | 23.0 ± 3.2 |  |
| Yes | 27 | 22.3 ± 2.6 |  |
| Mobile device |  |  | 0.58 |
| No | 1 | 24.0 ± 0.0 |  |
| Yes | 38 | 22.5 ± 2.8 |  |
| Web-enabled cell phone |  |  | 0.19 |
| No | 2 | 25.0 ± 0.0 |  |
| Yes | 37 | 22.4 ± 2.8 |  |
| Hours of Internet use (daily) |  |  | 0.55 |
| ≦3hours | 11 | 22.9 ± 3.4 |  |
| > 3 hours | 28 | 22.3 ± 2.5 |  |

Abbreviation: SD =standard deviation

**p* < 0.05.
