# Supplementary material for: The acceptance and impact of Google Classroom integrating into a clinical pathology course for nursing students: A technology acceptance model approach
Source: PLoS One. 2021 Mar 5;16(3):e0247819. doi: 10.1371/journal.pone.0247819 (PMC7935261; doi:10.1371/journal.pone.0247819)
Supplement: S2 Table — (DOCX) [file pone.0247819.s003.docx]

S2 Table. Pearson correlations between subscales of technological acceptance for the experimental group at the end-of-semester (n=39).

| Subscales | IQ | CSE | PP | PU | PEOU | ITU | Overall TA |
| --- | --- | --- | --- | --- | --- | --- | --- |
| Interaction quality (IQ) | 1.00 |  |  |  |  |  |  |
| Computer self-efficacy (CSE) | 0.56** | 1.00 |  |  |  |  |  |
| Perceived playfulness (PP) | 0.75** | 0.46** | 1.00 |  |  |  |  |
| Perceived usefulness (PU) | 0.66** | 0.42** | 0.67** | 1.00 |  |  |  |
| Perceived ease of use (PEOU) | 0.43** | 0.70** | 0.34* | 0.50** | 1.00 |  |  |
| Intention to use (ITU) | 0.68** | 0.56** | 0.76** | 0.72** | 0.36* | 1.00 |  |
| Overall technology acceptance (TA) | 0.85** | 0.76** | 0.84** | 0.82** | 0.67** | 0.86** | 1.00 |

***p* < 0.01; **p* < 0.05.
